# Predictors of loss to follow up among adult clients attending antiretroviral treatment at Karamara general hospital, Jigjiga town, Eastern Ethiopia, 2015: a retrospective cohort study

**DOI:** 10.1186/s12879-018-3188-4

**Published:** 2018-06-18

**Authors:** Wubareg Seifu, Walid Ali, Beyene Meresa

**Affiliations:** 1 College of Medicine and Health Sciences, Public Health department, Epidemiology and Biostatistics Unit, Jigjiga University, P.O. Box:1020, Jigjiga, Ethiopia; 2 Department of Radiology, Saint Paul’s Hospital Millennium Medical College, Addis Ababa, Ethiopia; 3 School of Public Health, College of Health Sciences, Mekelle University, Mekelle, Ethiopia

**Keywords:** Loss to follow up, ART, Jigjiga town, Predictors, Eastern Ethiopia

## Abstract

**Background:**

Retention in care and adherence to the treatment is very important for the success of the program while access for treatment is being scaled up. Without more precise data about the rate of loss to follow up as well the characteristics of those who disengage from the treatment appropriate interventions to increase ART adherence cannot be designed and implemented. Therefore the aim of this study was to determine incidence and predictors of loss to follow up among adult ART clients attending in Karamara Hospital, Jigjiga town, Eastern Ethiopia, 2015.

**Methods:**

An institutional based retrospective cohort study were undertaken among 1439 adult people living with HIV/AIDS and attending ART clinic between September 1, 2007 and September 1, 2014 at Karamara Hospital was undertaken. Loss to follow up was defined as not taking an ART refill for a period of 90 days or longer from the last attendance for refill and not yet classified as ‘dead’ or ‘transferred-out’. A Kaplan-Meier model was used to estimate rate of time to loss to follow up and Cox proportional hazards modeling was used to identify predictors of loss to follow up among ART clients.

**Result:**

Of 1439 patients, 830(58.0%) were females in their sex. The mean age of the cohort was 33.5 years with a standard deviation of 9.33. Around 213 (14.8%) patients were defined as LTFU. The incidence rate of loss to follow up in the cohort was 26.6% (95% CI; 18.1–29.6) per 100 person months. Patients with male sex [HR: 2.1CI;(1.3–3.4)], patients whose next appointment weren’t recorded [HR: 1.2, 95% CI; (1.12–1.36)] and patients who did not disclose their status to any one [HR: 2.8, 95% CI; (2.22–5.23)] were significantly associated with LTFU in the cox proportional model.

**Conclusion:**

Overall, these data suggested that LTFU in this study was high. The ART patients’ next appointment should be documented very well and as well the clients should be advised to adhere with treatment program as per the schedule. Defaulter tracing mechanism should be operational and strengthen in the health facility. Effective control measures should be designed for at-risk population such as male patients.

## Background

The rapid expansion on the utilization of antiretroviral therapy (ART) has transformed national Acquired Immune Deficiency Syndrome (AIDS) responses and as well contributed significant impact on the health of people living with human immunodeficiency virus (PLWHIV) [1]. The ART treatment has shown promising results in the reduction of HIV transmission and HIV/AIDS related morbidity and mortality. According to World Health Organization (WHO) report, ART has prevented an estimated 4.2 million deaths in Low and Middle Income Countries (LMICs) in the year 2013 [1].

At the beginning, most of the efforts to combat the AIDS epidemic were mainly focused on access to antiretroviral drugs. Patient adherence to the treatment and retention in care emerged as a critical concern for the health sector to gain the desired outcome of combined ART treatment regimen [1]. Loss to follow-up (LTFU) of clients from ART negatively impacts on the immunological benefits of ART, increase AIDS related morbidity, mortality and hospitalization. LTFU of patients from ART can result serious consequences such as discontinuation of treatment, drug toxicity, treatment failure due to poor adherence, and drug resistance [2–7].

Retention of patients in long term treatment programs has not given due attention since most large scale treatment providers have limited resources to trace missing patients. As a result much attention has focused on patient day-to-day adherence to antiretroviral treatment [4, 7–9]. Lost to follow up patients cannot easily reached out in most of the cases, because patients involved in such situation have decided to be out of care, either voluntarily or involuntarily. Providers typically do not know whether a lost patient has died, transferred to a new treatment site, unable to stay in care due to medical, economical, social or psychological barriers or simply chosen to discontinue their follow up [10].

Even though, Ethiopia has a 1.9% national HIV/AIDS prevalence [2, 11], the magnitude and predictors of LTFU after initiation of antiretroviral therapy are not well documented in most regions of the country. Without more precise data about the rate of loss to follow up as well as the characteristics of those who withdraw from the treatment appropriate interventions to increase ART adherence cannot be designed and implemented. Therefore, evidence based interventions that prevent LTFU in resource limited settings like Ethiopia will improve treatment outcomes and adherence in a cost effective approach.

## Methods

### Study setting

The study was conducted in Jigjiga town which is located at 638 Km from the capital city Addis Ababa. The total of population of the study area was 199, 756 according to 2007 census [2]. There were five governmental health institutions (3 health centers and 2 Hospitals) which provide ART service for the town and surrounding population. All diagnosed HIV positive individuals from VCT, PMTCT or inpatient services were sent to the ART center and registered. If they were eligible to start ART, a unique ART number was given to each patient. Once diagnosed with HIV/AIDS and eligible for ART, patients were provided with ART medication and care free of charge.

### Study design and participants

A retrospective observational cohort study was performed among all adult patients who initiated ART and were followed-up between September 1, 2007 and September 1, 2014 at Karamara General Hospital. All adult patients who initiated ART and were followed-up between September 1, 2007 and September 1, 2014 were included in this study. The study included all cases of lost-to-follow-up from governmental health institution ART clinics during the period of September 2007 to September 2014. Therefore, this was a census type study and didn’t involve any sampling. There were 2660 clients’ registered for ART service starting from September 2007 to September 2014 at Karamara General Hospital. Patients who were initiated on ART between the periods of September 2007 to September 2014 at karamara General Hospital were the source population for this study. All adult (18 years and above), non-pregnant, AIDS patients in the treatment program from September 2007 through September 2014 were eligible for the study. Patients were excluded if ART initiation or termination date was missing, and/or if dates were wrongly recorded such as, ART initiation date after ART termination date. As well all patients whose deaths were recorded on the medical files and patients who were started on ART but were transferred out to other health facility were excluded from the study.

### Measurement

The data for this research was secondary data collected routinely in the hospital for clinical monitoring and evaluation purposes and entered in an ART database during the follow-up time. The primary outcome variable was LTFU from ART follow-up care after initiation of treatment, confirmed by reviewing medical registration at the hospital, noted by ART adherence supporters. Data recording started from the date that patients started regular HIV care in the clinic to confirmation of a final event. All lost to follow up during September 1, 2007 and September 1, 2014 were included in the study. The patients’ identification numbers were used to generate the necessary sample from the records of the health facility for extracting data. Socio-demographic characteristics, baseline and follow up clinical and laboratory measurement information, and treatment outcomes were abstracted from patients’ cards. Data were retrieved from patients’ ART card by trained nurses working in the ART Clinic using uniform data abstraction format prepared for the study. Whenever relevant information was missing, the ART electronic database was consulted. The supervisors checked each completed questionnaire and principal investigator monitored the overall quality of the data collection. In this study lost to follow up was defined as if their last date of contact with the ART clinic was ≥3 months before the date of database closure and they were not known to have died or transferred out. Transferred-out was defined as if the patient had moved to another health facility with confirmed written documentation of transfer out. Proper recording was defined as when the health care provider stated the next appointment date and other related information of the ART clients in a written form on their charts. Whereas censored event was defined as at the date of death or transferred out or at the end of the study period.

### Data processing and analysis

Data were cleaned, edited, and entered onto Epidata version 3.2.1 and exported to SPSS windows version 20 and STATA version 10 for further analysis. Descriptive statistics such as median, mean, SDs and percentage were used to investigate the characteristics of the cohort. Person years of follow up were calculated by assessing the date of enrollment for ART and lost follow up or censoring. Survival analysis and the Kaplan-Meier test were used to investigate factors that influence time to loss to follow up. Hazard ratios (HR), with 95% confidence intervals were used as effect measures. A *p*-value of 0.05 was considered as significant. Kaplan Meier curves were plotted and compared using log rank tests. Ethical approval was obtained from Jigjiga University ethical review board committee. In addition an official support letter was obtained from the medical director of the Hospital and the ART department as well all collected data were kept confidential. While reviewing patients’ records, non-personal identifiers such as patients’ medical registration number were used to distinguish study participants during data collection. Written informed consent from study participants were not feasible since this was analysis of secondary data retrieved from the patient cards/database which is available in the Hospital. Because of this reason the ethical committee of the university waived written consent from the study participants directly. The hospital database management department had consented each HIV/AIDS patients to use the data for research purpose through confidential way at the time enrolment to the ART program.

## Results

**Fig. 1:**
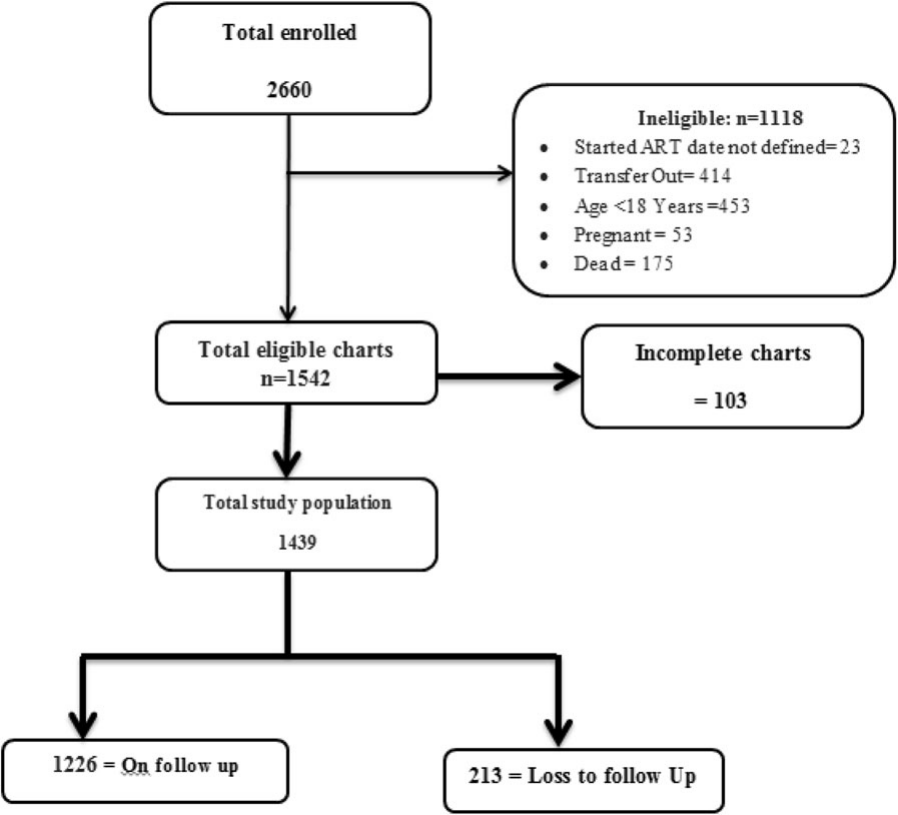
Profiles of HIV/AIDS clients at Karamara Hospital, Jigjiga (September 1, 2007 to September 1, 2014, Ethiopian Somali Regional State, Eastern Ethiopia; 2015

Between September 1, 2007 and September 1, 2014, a total of 2660 HIV/AIDS patients were newly enrolled for HIV care at Karamara General Hospital ART clinic. Of these, 1542 were eligible for this study based on the inclusion criteria. Of the eligible patients, 103 (6.7%) of adult ART patients were excluded from the study because their charts were incomplete. Out of the remaining 1439 study population, 1226 (85.2%) were patients who were under active follow-up for their HIV care while the other 213 (14.8%) were lost to follow-up during the ART period (Fig. 1).

### Baseline socio-demographic characteristics of the cohort

In this study a total of 1439 adult patients on ART between September 2007 and 2014 were included in the statistical analysis. These adult ART patients were followed for about 10,755 person-months. Out of the 1439 study participants analyzed in in this study, 830(58.0%) were females by their sex. By the time of enrollment into HIV/AIDS care, majority 377 (26.2%) of the study subjects were between 28 and 32 years of age while 275 (19.1%) were between the age group of 23–27 years of age with a mean (± SD) age of 33.49(±9.33) years. Of the total study population, 680 (47.3%) were married. With regard to educational status of study participants, 351 (37.3%) of patients completed primary school, followed by 487 (33.8%) of patients with no education at all. Majority 713 (49.5%) of study participants were Orthodox Christianity by their religion. Just over one quarter 181 (12.6%) of the study participants enrolled in HIV/AIDS care were lived outside Jigjiga town (Table 1).

**Table 1:** Baseline socio-demographic characteristics of adult ART patients at Karamara Hospital from September 1, 2007 to September 1, 2014), Somali region, Jigjiga town, Eastern Ethiopia, 2015

Variables	Frequency	Percentage (%)
Sex		
Male	605	42.0
Female	834	58.0
Religion		
Muslim	658	45.7
Orthodox	713	49.5
Protestant	60	4.2
Others	8	0.6
Marital status		
Married	680	47.3
Never married	211	14.7
Divorced	391	27.2
Widowed	142	9.9
Separated	15	1.0
Age group		
18–22	122	8.5
23–27	275	19.1
28–32	377	26.2
33–37	237	16.5
38–42	204	14.2
> = 43	224	15.6
Place of Residence		
Jigjiga	1258	87.4
Out of Jigjiga	181	12.6
Educational status		
No education	487	33.8
Primary level	552	38.5
Secondary and above	388	27.0
Employment status		
Government	205	14.2
House wife	116	8.1
Daily laborer	407	28.3
Unemployed	579	40.2
Others	132	9.1
Contact Person		
Yes	1081	75.1
No	358	24.9

### Baseline clinical characteristics of the cohort

Among the 1439 patients who had their baseline CD4 cell count documented, 935 (65%) had CD4 count ≤200 cell/μl while 113 (7.9%) of them had a CD4 count > 350 cell/μl. The median initial CD4 count was 166 cell/μl (IQR = 83 to 233 cell/μl). Among the 1439 charts that had complete information about WHO staging, majority 670 (46.6%) of patients were stage III followed by 328 (22.8%) stage I patients. Among 1439 patient charts that had documentation on past history of tuberculosis, 367 (25.5%) patients had a history of tuberculosis treatment. Most 811 (56.4%) of the study population have a functional status followed by ambulatory 488 (33.9%) state whereas most 546 (36.9%) of the ART clients discloses their status to their family members (Table 2).

**Table 2:** Baseline clinical characteristics of adult ART patients at Karamara Hospital from September 1, 2007 to September, 2014, Somali region, Jigjiga town, Eastern Ethiopia, 2015

Variables	Frequency	Percentage (%)
CD4 cell count/μl		
<=200	935	65.0
201–250	195	13.6
251–300	196	13.6
301–350	113	7.9
> 351	113	7.9
WHO stage		
Stage I	328	22.8
Stage II	228	15.8
Stage III	670	46.6
Stage IV	213	14.8
History of TB treatment		
Yes	367	25.5
No	1072	74.5
Next appointment recorded		
Yes	1092	75.9
No	347	24.1
Base line functional status		
Functional	811	56.4
Ambulatory	488	33.9
Bed ridden	140	9.7
ART regimen substitutions		
Yes	172	12.0
No	1267	88.0
Disclosure status		
Family members	546	36.9
Spouse only	453	30.6
No one	358	24.2
Relative/friend	122	8.1

### The incidence of loss to follow up

**Fig. 2:**
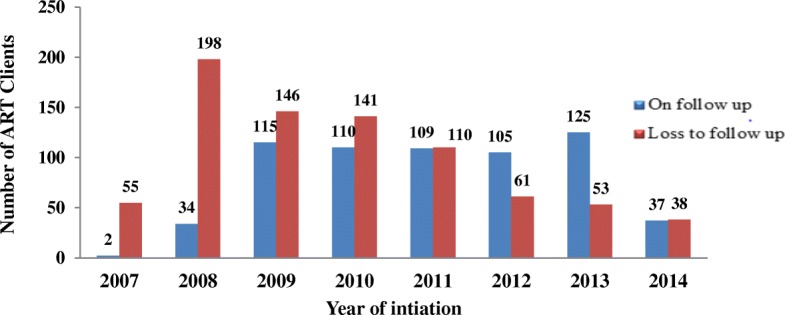
Number of clients retained and loss to follow up from ART program by each year at Karamara General Hospital, from September 1, 2007 to September, 2014, Ethiopian Somali regional State, Eastern Ethiopia, 2015

**Fig. 3:**
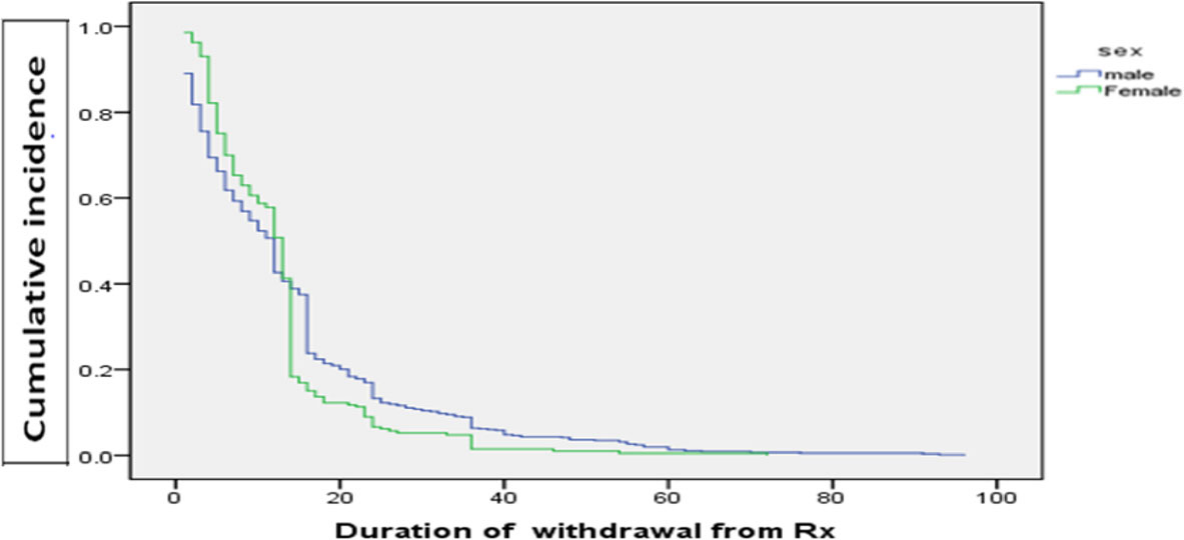
Kaplan-Meier estimation of cumulative incidence of loss to follow up by sex among adult ART attendants at Karamara Hospital, Jigjiga, Ethiopia, 2015

The overall incidence rate of LTFU in the cohort was estimated to be 26.6% (95% CI; 18.1–29.6) per 100 person months. Patient retention in care was 14.7% at 2 years and 49.8% at 5 years in the cohort. Approximately 96.5% (55/57) of the patients that became lost to follow up were lost within the first year of being initiated on ART. The absolute number of patients that were LTFU with each year on ART decreased over time (Fig. 2). The median time on ART before discontinuation of follow-up was 12 months (IQR = 4 to 16 months). The cumulative incidence of loss to follow up among male ART clients is higher than the female ones (Fig. 3).

### Risk factors associated with LTFU among adult ART clients

In the multivariate cox regression model patients with male sex [HR: 2.1CI; (1.3–3.4)], patients whose next appointment were not properly recorded [HR: 1.2, 95% CI; (1.12–1.36)] and patients who did not disclose their status to any one [HR: 2.8, 95% CI; (2.22–5.23)] were significantly associated with LTFU in the cox proportional hazard model (Table 3).

**Table 3:** Cox regression analysis of factors associated with LTFU among adult patients on ART therapy at Karamara General Hospital, September 2007 to September 2014, Somali region, Jigjiga town, Eastern Ethiopia, 2015

Variables	Loss to Follow Up		AHR	*P*- Value
	Yes	No	(95% CI)	
Sex				
Male	107 (34.2)	498 (65.8)	2.1 (1.3–3.4)	0.034*
Female	106 (24.7)	728 (75.3)	1	
Next appointment recorded properly				
Yes	190 (17.4)	902 (82.6)	1	0.000*
No	23 (6.6)	324 (93.4)	1.2 (1.12–1.36)	
History of TB treatment				
Yes	71 (19.4)	296 (80.6)	1.2 (0.08–1.38)	
No	142 (13.2)	930 (86.8)	1	0.561
WHO Stage^a^				
I	38 (11.6)	290 (88.4)	1	
II	29 (12.7)	199 (87.3)	0.4 (0.34–2.34)	0.341
III	111 (16.6)	559 (83.4)	0.8 (0.40–1.17)	0.123
IV	35 (16.4)	178 (83.6)	0.6 (0.28–1.43)	0.112
Functional status^b^				
Functional	81 (10.0)	730 (90.0)	1	
Ambulatory	96 (19.6)	392 (80.4)	0.73 (0.44–1.2)	0.2213
Bed ridden	36 (25.7)	104 (74.3)	1.31 (0.86–1.9)	0.2364
CD4 Count				
= < 200	152 (16.3)	783 (83.7)	0.7 (0.5–1.0)	0.117
201–250	21 (10.8)	174 (89.2)	0.6 (0.4–1.8)	0.712
251–300	16 (8.2)	180 (91.8)	0.7 (0.6–1.0)	0.371
301–350	10 (8.9)	103 (91.1)	0.9 (1.1–1.8)	0.682
> = 351	14 (12.4)	99 (87.6)	1	
Disclosure status				
Disclosed to any one	76 (7.0)	1005 (93.0)	1	
Not disclosed to any one	137 (38.3)	221 (61.7)	2.8 (2.22–5.23)	0.034*

Note

*statistically significant at *p*-value < 0.05

^a^is based on the clinical sign and symptom complex

^b^Functional = able to perform usual work in or out of the house, harvest, go to school

Ambulatory = able to perform activities of daily living but not able to work or play

Bedridden = not able to perform activities of daily living

## Discussion

This study attempted to determine the incidence and predictors of loss to follow up from ART treatment program among adult population. The cumulative incidence of LTFU was 14.8% among adult ART attendants. In the multivariate analysis being male, next appointment not properly recorded on the medical chart and failed to disclose HIV/AIDS status to any one were the independent predictors of loss to follow up among adults aged 18 years and above.

The overall incidence rate of loss to follow up was 14.8% in this study. This finding is lower than studies conducted in Sub Saharan countries (20–40%) [12, 13] but similar to analogous studies done in Northwest Ethiopia, Bahrdar Feleg Hiwot Hospital and Gonder Referral Hospital which showed that the lost to follow up rate were 8.4, 18 and 19% respectively [14–16].

Generally we had found a progressive decrease in the incidence of LTFU patients with each year after initiation of ART. The incidence of loss to follow up in our study was found to be 85.3, and 50.2% by the end of the 2nd and 5th year respectively. This finding is contrast with other studies finding whereby there is progressive increase in LTFU overtime [17–23]. This difference might be attributed due to decentralization of care from referral centers to primary health care facilities in the study area. Currently the Ethiopian health care delivery approach gives much emphasis for decentralization of ART cares to the lower health facilities. This has been largely attributed to the fact that decentralized clinics tend to be closer to patients homes requiring them to travel less, reducing transport costs and building stronger links between health services and the community. The closer proximity can also make tracing more feasible from decentralized clinics [24–26]. Additionally the enhancement of community outreach services through several mechanisms, such as involving community health workers, recording detailed information of each patient and expanding the peer outreach program might be the possible reasons for the improvement of retention over time in among the study population.

In this study males were [AHR: 2.1 95% CI (1.3–3.4)] found to be at higher risk of LTFU from adult ART treatment programs. This finding is in line with studies conducted in Northwest Ethiopia, Oromia region, and India [16, 27, 28]. The observed difference might be attributed to variations in the life styles as men spent much time away from their home coupled with high risk of drug abuse might leads to discontinuation from the treatment programs compared to their counter parts. Moreover, most 883 (61.4%) of the study participants enrolled with advanced stage diseases (III & IV) were males which makes it highly likely that they were lost to follow-up. This finding is consistent with other studies which reported that HIV-infected men seek treatment lately compared to women and are therefore at greater risk of an adverse clinical outcome including death [29, 30]. Additionally gender based norms imposed by the society where by men is considered as the symbol of strength and proud suppress their health care service demands [31, 32]. As a result, they might neglect their weaknesses and susceptibility before achieving the expected level of treatment adherence to ART. This implies that HIV/AIDS treatment response and follow-up mechanism should be gender sensitive in order to prevent loss to follow up.

Study participants without a proper record of the next visit date had a high chance of being LTFU from ART treatment [HR: 1.23, 95% CI (1.12–1.36)] as compared to their counterparts. This finding was supported by studies which were conducted in Northwest Ethiopia, Malawi and Kenya [33, 16, 34]. This could be because a clinician who failed to record the next appointment date on the patient’s chart might also forgot to provide written or verbal information for the patient about the specific date of the next visit and other related messages as well. Hence the probability of return back to the ART clinic for such kind of clients will be unlikely.

Clients who did not disclose their HIV/AIDS status were 1.8 more likely to withdraw from the treatment program as compared to their counterparts [AHR: 2.8, 95% CI (2.22–5.23)]. This finding is consistent with studies conducted in South Africa and India [35, 36]. This might be attributed that clients will adhere much more if they disclose their status since ART treatment adherence needs comprehensive support and care from different part of the community as well. In this study CD4 count, advanced WHO stage (III/IV) and functional state were not significantly associated with LTFU unlike the findings of studies elsewhere [14–16]. This difference might be due to difference in study setting and study population as well.

This study has some limitations that may impact interpretation of our findings. The first limitation was resulted from the poor tracing and documentation system of patients in the ART program. Patients, who were labeled as LTFU might have been died or self-referred to other facilities. This could have led to an over-estimation of the incidence rate of LTFU in our study The second limitation was missing data with regard to baseline socio-demographic and clinical variables. Following this we only analyzed 54% of the total ART enrolled clients during the study period and this might under or overestimate the incidence of LTFU in our study. Despite these limitations, we believe that our study findings will provide an important insight into the magnitude and the factors related to LTFU among patients on ART.

## Conclusion and recommendations

The cumulative incidence of loss to follow up among adult population in this study was found 14.8%. Among the socio-demographic factors sex were significantly associated with LTFU among adults ART attendants. While next appointment recording status and disclosure status were significantly associated with LTFU among the clinical factors. The Hospital ART monitoring and follow up department should strengthen and make the clients tracing system so as to increase client retention in the facility back. On top of this the ART department should record the clients next appointment time and advise them to return back accordingly. Additionally effective control measures should be designed for at-risk population such as male ART clients by ART department of the hospital.

## Abbreviations

AHR: Adjusted Hazard Ratio
AIDS: Acquired Immune Deficiency Syndrome
AIDS: Acquired Immune Deficiency Syndrome
ART: Anti-retroviral Therapy
CHR: Crude Hazard Ratio
CI: Confidence Interval
DRCS: Directorate of Research and Community Service
EDHS: Ethiopian Demographic and Health Survey
FMoH: Federal Minister of Health
HAPCO: HIV/AIDS Prevention and Control Office
HIV: Human Immunodeficiency Virus
HR: Hazard Ratios
IQR: Inter quartile range
LMIC: Low and Middle Income Countries
LTFU: Loss to follow up
PLWHIV: Peoples Living with Human Immunodeficiency Virus
PMTCT: Prevention of Mother to Child Transmission
SD: Standard Deviation
SPSS: Statistical Package for Social Science
SSA: Sub Saharan Africa
VCT: Voluntary Counseling and Testing
WHO: World Health Organization
